# MFCA-Net: a deep learning method for semantic segmentation of remote sensing images

**DOI:** 10.1038/s41598-024-56211-1

**Published:** 2024-03-08

**Authors:** Xiujuan Li, Junhuai Li

**Affiliations:** 1 School of Computer Science and Engineering, Xi’an University of Technology, Xi’an, 710048 China; 2 School of Information, Xi’an University of Finance and Economics, Xi’an, 710100 China

**Keywords:** Computer science, Information theory and computation

## Abstract

Semantic segmentation of remote sensing images (RSI) is an important research direction in remote sensing technology. This paper proposes a multi-feature fusion and channel attention network, MFCA-Net, aiming to improve the segmentation accuracy of remote sensing images and the recognition performance of small target objects. The architecture is built on an encoding–decoding structure. The encoding structure includes the improved MobileNet V2 (IMV2) and multi-feature dense fusion (MFDF). In IMV2, the attention mechanism is introduced twice to enhance the feature extraction capability, and the design of MFDF can obtain more dense feature sampling points and larger receptive fields. In the decoding section, three branches of shallow features of the backbone network are fused with deep features, and upsampling is performed to achieve the pixel-level classification. Comparative experimental results of the six most advanced methods effectively prove that the segmentation accuracy of the proposed network has been significantly improved. Furthermore, the recognition degree of small target objects is higher. For example, the proposed MFCA-Net achieves about 3.65–23.55% MIoU improvement on the dataset Vaihingen.

## Introduction

Remote sensing technology is widely used in various fields such as urban planning^1,2^, land resource utilization^3–5^, and precision agriculture^4,6^. The semantic segmentation technique is an important research direction of RSI. Various semantic segmentation methods have been developed and applied in practical applications. The threshold-based image segmentation method^7,8^ realizes semantic segmentation by classifying the image gray histogram using different gray thresholds. The edge-based segmentation method was used by Roberts^9^, Sobel^10,11^, Prewitt ^12,13^, and other edge detection operators^14,15^ in identifying and connecting the boundary pixels to form the contour of the edge. The image region segmentation method classifies the pixels and creates regions based on their similar characteristics, and methods such as region production and split merge are frequently employed^16–18^. Traditional semantic segmentation approaches mentioned above need to set parameters manually, and the segmentation accuracy is low. In addition, they cannot adapt to image segmentation tasks with a large amount of semantic information.

In recent years, deep learning has achieved profound success in remote sensing image applications^19–21^, especially in semantic segmentation^22–24^. Zheng et al.^25^ applied the U-Net^26^ model widely used in medical image segmentation to RSI and trained on the GF-2 RSI dataset. Xuan et al.^27^ suggested a multipath encoder structure for extracting the features to improve target object boundary classification accuracy in RSI. Zheng et al.^28^ developed a semantic segmentation model using spatial context acquisition of the Markov random field model to enhance the segmentation accuracy of different land categories. Sun et al.^29^ proposed an improved U-Net network that groups channels in a multitasking manner and processes heterogeneous image segmentation through information fusion. Chen et al.^30^ presented an improved network framework for RSI semantic segmentation based on the spatial channel fusion compression and excitation module. Fan et al.^31^ improved DeepLab^32^ for extracting cultivated land information, introducing a parameter to adjust the dilated convolution kernel and adding a more precise decoder group to the model structure. Wang et al.^33^ used ResNet-34^34^ as the backbone and built a double-branch encoder to extract lakes and water bodies from the Qinghai Tibet Plateau.

Transformer is a deep learning model based on the self-attention mechanism. Since the transformer captures long-distance dependencies between local and global features by comparing their correlations at all spatial positions, it has more robust modeling capabilities. Therefore, more and more researchers are applying it to computer vision tasks. Zhang et al.^35^ propose a semantic segmentation model using a transformer as the backbone network to obtain better remote spatial dependencies. Wang et al.^36^ combine Swin Transformer with Densely Connected Feature Aggregation Module to propose a new semantic segmentation model for remote sensing images.

Generative Adversarial Networks (GANs)^37^ belong to generative models. Luc et al.^38^ first introduced GANs into image semantic segmentation. Due to the high time and money costs of large-scale annotated datasets, many researchers have shifted their research direction to GAN-based semantic segmentation. Li et al.^39^ propose a distribution-aligned semantic segmentation network based on GAN. Ma et al.^40^ suggest a novel GAN network, which integrates additional discriminators to learn domain-specific features and captures cross-domain dependencies of semantic feature representations through mutually enhancing attention transformers. Algorithms based on GANs can generate samples and determine their authenticity, but their performance could be better for large-scale training.

In summary, the feature learning ability of neural networks mentioned above has shown substantial advantages in the semantic segmentation of RSI. However, RSI is prone to the problem of unbalanced sample classification, or there may be significant differences in classification sizes. These characteristics result in insufficient network, classification errors, and missed detection of small target objects, decreasing overall segmentation accuracy. This paper presents a new deep neural network for remote sensing image segmentation in response to the above issues. The main contributions of this study can be summarized as follows:

- A new neural network, MFCA-Net, is proposed for the semantic segmentation of RSI. Moreover, the results of the proposed MFCA-Net are superior to those of other approaches under limited training sample scenarios.
- In IMV2, attention mechanisms are introduced in the shallow and deep feature maps respectively to improve the segmentation accuracy of the network.
- The MFDF module obtained a more extensive range of contextual information and denser feature sampling points, effectively solving the problems of unbalanced sample classification and low segmentation accuracy of small target objects.

## Methods

**Figure 1:**
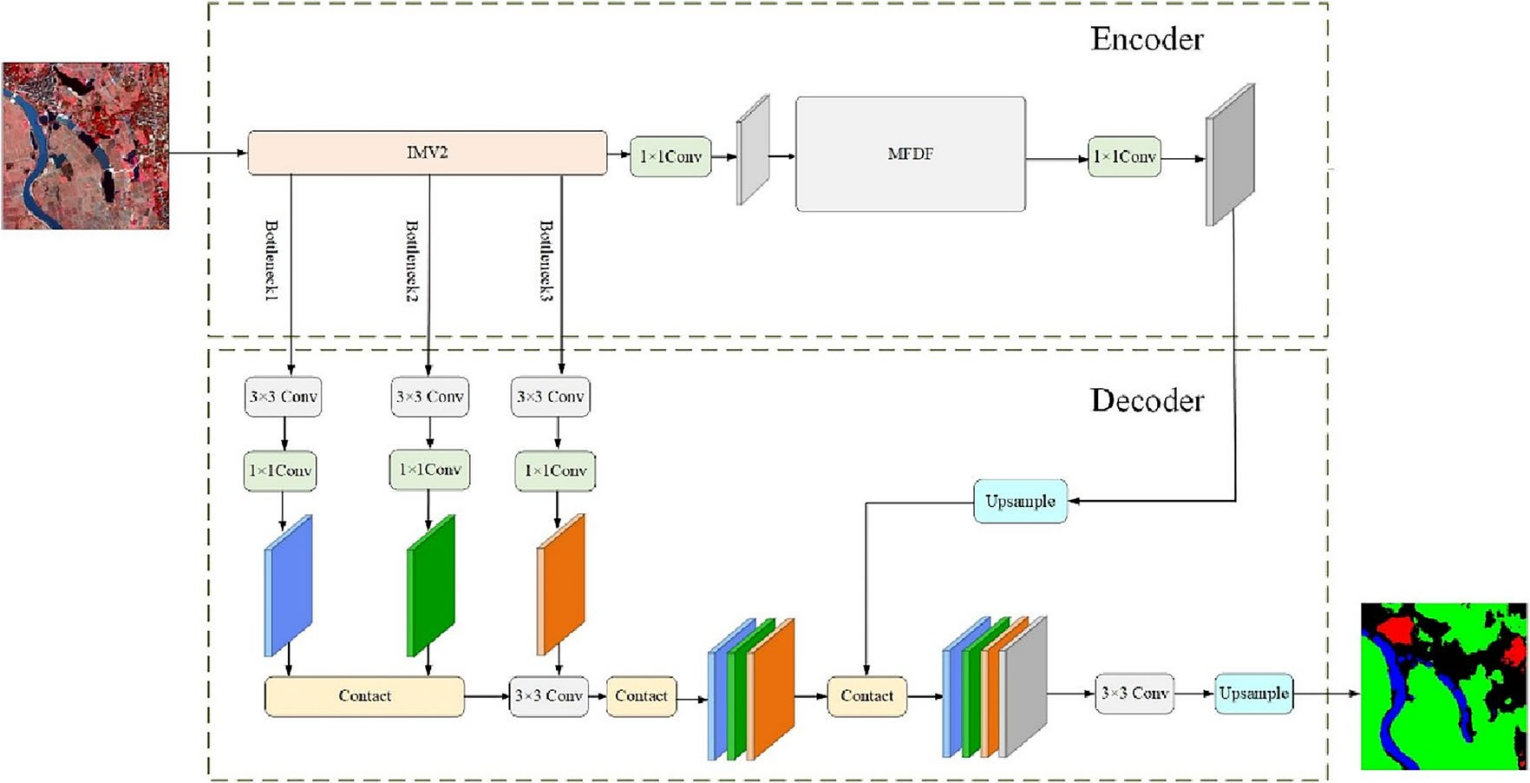
The overall architecture of the proposed MFCA-Net network (this figure was drawn by Visio 2021, which can be available at https://www.microsoftstore.com.cn/software/office/visio-standard-2021).

The overall framework of MFCA-Net adopts an encoding–decoding structure, as shown in Fig. 1. We introduce MobileNet V2^41^ as the backbone and improve it. The attention mechanism is used in the shallow and deep feature layers. We add the MFDF module, which not only obtains a larger receptive field but also attempts to solve the problem of identifying small sample targets through denser sampling points. In decoding, three branches are introduced from the feature extraction module, fused, and then upsampled to achieve pixel-level classification of RSI.

### Encoder

#### IMV2

The feature extraction module uses the lightweight MobileNet V2 to ensure the learning performance and efficiency of the network. Based on depthwise and pointwise convolution, the parameter quantity of MobileNet V2 is only 1/9 to 1/8 of the standard convolution. Nevertheless, all channels in the feature map are assigned the same weight in MobileNet V2. We improve it and introduce channel attention mechanisms (CA) after the shallow feature map Bottleneck1 and deep feature map Bottleneck6, respectively. The operation process of CA includes compression, activation, and scale operations.

##### Compression operation

Firstly, the feature map is global pooled. Then, the feature vector is compressed into a one-dimensional vector through the convolution and batch normalization (BN) layers. Each dimension of the one-dimensional vector represents the weight of each channel. The operation can be expressed as follows:

1 $${\text{z}}={{\text{F}}}_{{\text{sq}}}\left({\text{f}}\right)=\frac{1}{{\text{H}}\times {\text{W}}}\sum_{{\text{i}}=1}^{{\text{H}}}\sum_{{\text{j}}=1}^{{\text{W}}}{\text{f}}\left({\text{i}},{\text{j}}\right),$$

where ${{\text{F}}}_{{\text{sq}}}$ is the compression operation function, ${\text{f}}\in {{\text{R}}}^{{\text{H}}\times {\text{W}}}$ is a set of two-dimensional feature maps; ${\text{f}}\left({\text{i}},{\text{j}}\right)$ is one of the elements, H and W are the height and width of the feature map, respectively; z is the output of compression operation.

##### Activation operation

The feature map vector’s channel dimension is reduced to the original 1/r through the first full connection layer (FC1), resulting in a 1 × 1 × C/r feature map shape, and r expresses the dimensionality reduction ratio. After that, Funnel activation (FReLU)^42^ performs the nonlinear processing. The activation function in the MobileNet series, whether Relu or Relu6, models the one-dimensional linear space of the pixel itself, so it is easy to lose the characteristics of the pixels around the center point and reduce the model learning ability. FReLU uses funnel conditions to obtain the maximum value between the center point and the states. The formula is as follows:

2 $$FReLU={\text{max}}\left({{\text{x}}}_{{\text{c}},{\text{i}},{\text{j}}},{\text{T}}\left({{\text{x}}}_{{\text{c}},{\text{i}},{\text{j}}}\right)\right),$$

where ${{\text{x}}}_{{\text{c}},{\text{i}},{\text{j}}}$ is the pooling window centered with position $({\text{i}},{\text{j}})$ on channel C. ${\text{T}}\left({{\text{x}}}_{{\text{c}},{\text{i}},{\text{j}}}\right)={{\text{x}}}_{{\text{c}},{\text{i}},{\text{j}}}^{{\text{w}}}\cdot {{\text{p}}}_{{\text{c}}}^{{\text{w}}}$, ${{\text{p}}}_{{\text{c}}}^{{\text{w}}}$ is the parameters shared by this window in the same channel. Therefore, a funnel-shaped two-dimensional feature extractor can obtain more abundant image context feature information, which helps improve the segmentation accuracy. The feature map of the feature map vector is raised back to the channels’ original number through the second full connection layer (FC2). Additionally, it is transformed into a normalized weight vector, with values varying between 0 and 1, using a sigmoid function.

##### Scale operation

The normalized weight and the original input characteristic map channel are multiplied to generate the weighted distinct map. The formula is

3 $${\text{x}}={{\text{F}}}_{{\text{scale}}}\left({\text{f}},{\text{s}}\right)={\text{s}}\cdot {\text{f}}\left({\text{i}},{\text{j}}\right),$$

where ${{\text{F}}}_{{\text{scale}}}$ is the scale operation; ${\text{x}}$ is a value in the last output X of the attention module; ${\text{X}}=\left[{{\text{x}}}_{1},{{\text{x}}}_{2},\ldots ,{{\text{x}}}_{{\text{c}}}\right]$.The entire process is a parameter learnable process. The contribution weights of different channels are obtained through backpropagation training. The structure diagram of IMV2 is given in Table 1.

**Table 1:** The structure of IMV2.

Input	Operator	t	c	n	s
512^2^ × 3	conv2d	–	32	1	2
256^2^ × 32	Bottleneck1 + CA	1	16	1	1
256^2^ × 16	Bottleneck2	6	24	2	2
128^2^ × 24	Bottleneck3	6	32	3	2
64^2^ × 32	Bottleneck4	6	64	4	2
32^2^ × 64	Bottleneck5	6	96	3	1
32^2^ × 96	Bottleneck6 + CA	6	160	3	2
16^2^ × 160	Bottleneck7	6	320	1	1
16^2^ × 320	conv2d	–	1280	1	1
16^2^ × 1280	avgpool	–	–	1	–
1 × 1 × 1280	conv2d	–	K	–	

In Table 1, $t$ is the expansion factor; $c$ is the depth of the output characteristic matrix; $n$ is the number of iterations of bottleneck; ${\text{s}}$ is the step length.

#### MFDF

**Figure 2:**
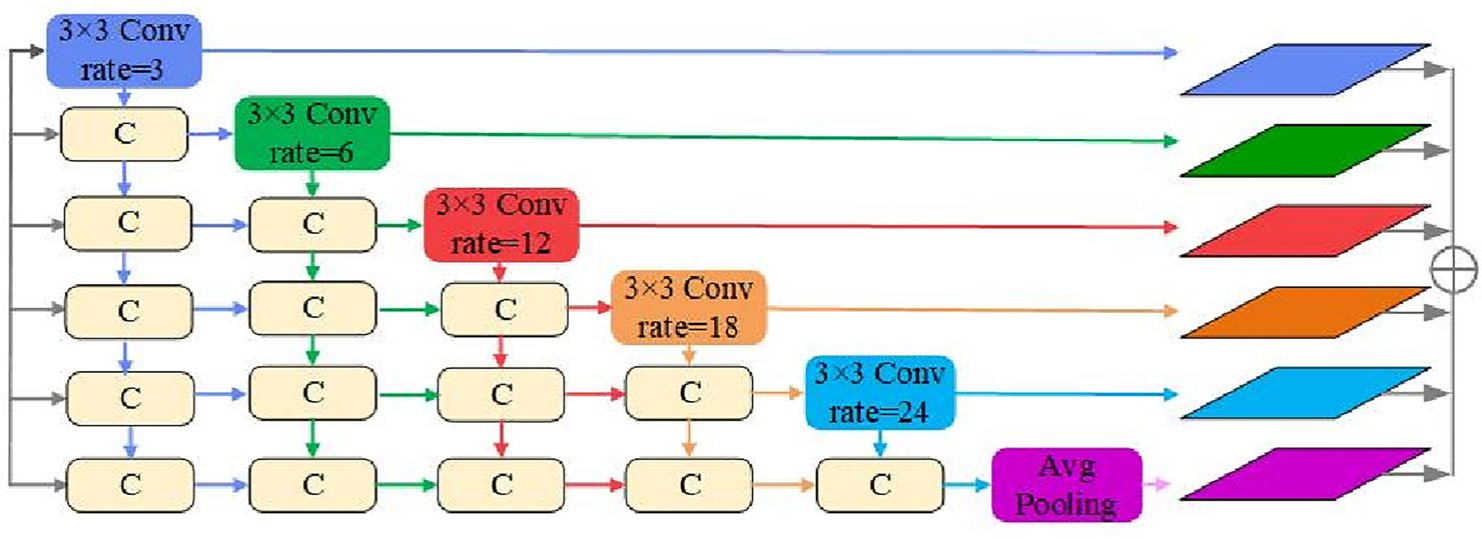
The structure of MFDF, and c represents concatenation operation (this figure was drawn by Visio 2021, which can be available at https://www.microsoftstore.com.cn/software/office/visio-standard-2021).

The atrous spatial pyramid pooling (ASPP) proposed by DeepLab V2^43^ contacts feature maps with different dilation rates. Although this method can get a larger receptive field, it is only effective for some large objects, and fewer sampling points can be captured for fewer categories and small target objects. The design of MFDF aims to address the above issues. The study fuses the convolution feature maps of 3, 6, 12, 18, and 24 with various dilation rates. Adaptive average pooling can integrate a broad range of spatial information and prevent overfitting, so adaptivepool2d is added to this module. These six branches are densely connected backward, and the overall MFDF structure is depicted in Fig. 2.

Each dilation layer can be represented as follows:

4 $${{\text{x}}}_{{\text{l}}}={{\text{H}}}_{{\text{K}},{{\text{d}}}_{{\text{l}}}}\left(\left[{{\text{x}}}_{{\text{l}}-1},{{\text{x}}}_{{\text{l}}-2},\dots ,{{\text{x}}}_{0}\right]\right),$$

where ${{\text{d}}}_{{\text{l}}}$ is the dilation rate of one layer; […] is the splicing operation of the feature layer; $\left[{{\text{x}}}_{{\text{l}}-1},{{\text{x}}}_{{\text{l}}-2},\dots ,{{\text{x}}}_{0}\right]$ is the output of all layers before splicing. For the expanded convolution layer with dilation rate d and convolution kernel size K, the receptive field size is computed as follows:

5 $$R=\left(d-1\right)\times (K-1)+K$$

Stacking the two convolution layers together can provide a large receptive field. If two convolution layers with convolution kernels K_1_ and K_2_ are superimposed, the new receptive field is

6 $$K={K}_{1}+{K}_{2}-1.$$

The above formula indicates that the receptive field of the densely connected characteristic map is 128. In contrast, the receptive field of the ASPP with the same void rate is only 51, i.e., the receptive field of MFDF is more than twice as large as that of the ASPP.

### Decoder

Relevant studies^44,45^ have indicated that increasing the fusion of shallow feature maps containing details can improve segmentation accuracy. The present research enhances the application of low-level feature maps. After 3 × 3 and 1 × 1 convolution to adjust the channels of feature maps, bottleneck1, and bottleneck2 perform fusion operations, then achieve downsampling using convolution with stride 2. After fusing with bottleneck3, the feature maps combine with the deep feature map. Bilinear interpolation of four times is performed for upsampling to produce the segmentation image.

### Loss function

The loss function often used in semantic segmentation is cross-entropy loss, which assigns equal weight to all categories. The present study adds weight factors to the loss function to improve the importance of a few classes in the loss function and balance the distribution of the loss function. It uses the focal loss function^46^. The formula is as follows:

7 $${L}_{FL}\left({p}_{t}\right)=-{\alpha }_{t}{\left(1-{p}_{t}\right)}^{\gamma }log{p}_{t}^{\prime},$$

where α is a category balance parameter used to adjust the category balance degree; γ is the focusing parameter used to focus complex samples; Pt is the probability value of the prediction category. Experiments revealed that weight adjustment slightly improves the result.

## Experiments

We design two experiments to verify the performance of the proposed MFCA-Net: (i) an experimental investigation of the superiority of the proposed approach over six state-of-the-art methods, namely, SegNet^47^, U-Net^26^, PSPNet^48^, DANet^49^, DeepLab V3+^50^, and A2-FPN^51^. SegNet proposed an unpooling structure that applied the max pooling index, improving the recognition of segmentation boundaries. U-Net is an entirely symmetric semantic segmentation model. The first half of its structure is feature extraction, and the second half is upsampling. PSPNet introduces a pyramid pooling module to capture contextual information at different scales, thereby improving semantic segmentation performance. The DANet model introduces both position and channel attention, downsampling using ResNet as the backbone network, reducing it from 32 to 8 times while retaining more detailed information to improve segmentation performance. DeepLab V3+ uses atrous spatial pyramid pooling to concatenate feature maps obtained through convolution operations with different void ratios, achieving multi-scale feature extraction. The A2-FPN model performs semantic segmentation of fine-resolution remote sensing images by adding an attention aggregation module to the feature pyramid network. (ii) An ablation experiment given promoting the widespread use of the proposed MFCA-Net.

The present study uses pixel accuracy (PA), mean PA (MPA), mean intersection over union (MIoU), and frequency-weighted intersection over union (FWIoU) to determine segmentation accuracy. The operating system of this experiment is Windows 10, the graphics card is NVIDIA Geforce RTX3060, the Cuda version for parallel computing architecture is 11.0, and the deep learning framework is Pytorch 1.7.

### Dataset description

Two datasets: Vaihingen (https://www.isprs.org/education/benchmarks/UrbanSemLab/2d-sem-label-vaihingen.aspx) and Gaofen Image Dataset (GID) (https://www.cvmart.net/dataSets/detail/765?channel_id=op10&utm_source=cvmartmp&utm_campaign=datasets&utm_medium=article) are used to assess the effect of MFCA-Net. The Vaihingen dataset is collected by airborne imaging equipment of aerial vehicles, and the image collection location is the small village of Vaihingen in Germany. The data imaging consists of three bands: near-infrared, red, and green. The average resolution is 2494 × 2064, and the dataset is trimmed to a fixed size of 512 × 512 using 75% interblock coverage. 3300 images are obtained using horizontal and vertical flip and rotation operations to enhance the image. The dataset includes six classifications: impermeable surfaces, buildings, low vegetation, trees, cars, and backgrounds. The proportions of these six categories are 27.8%, 26%, 22.9%, 21.3%, 1.2%, and 0.8%, respectively, showing the category imbalance problem.

GID is a large-scale, high-resolution, remote-sensing, land-cover image dataset based on China's Gaofen-2 satellite data. These images were taken from over 60 cities in China, and each image is clear and of high quality, without any cloud or fog obstruction. The GID dataset has a vibrant spectrum, texture, and structure diversity, which is very close to the natural distribution characteristics of land features. GID includes 10 images with a spatial resolution of 4 m and an image size of 6908 × 7300 pixels. High interclass similarity and low intraclass discrimination are characteristics of GID images. Similarly, 31,500 images with the size of 512 × 512 are obtained after data enhancement methods. In light of the large dataset, the present study randomly selects 5000 images to create a small dataset. The dataset is classified into six categories: background, buildings, cultivated land, woodland, grassland, and water. The problem of sample unbalance is also apparent. The proportion of grassland is tiny, only 1.6%. Except for the background, the proportion of cultivated land is the highest, close to 30%.

## Quantitative comparison and visual performance

### Experiments on Vaihingen

Table 2 lists the Vaihingen test set and highlights the best performance in bold. The experimental results show that the segmentation accuracy of DANet, DeepLab V3+, and A2-FPN models is similar. The A2-FPN model proposed in 2022 has higher segmentation accuracy. MFCA-Net is the highest in all other metrics except for being less than 1% lower than DeepLab V3+ in MPA metrics. Compared to A2-FPN, MIoU and FWIoU indicators are 3.18% and 2.86% higher, respectively.

**Table 2:** Results on Vaihingen.

Method	PA	MPA	MIoU	FWIoU
SegNet^47^	78.79	63.91	53.22	65.03
U-Net^26^	83.23	66.42	56.49	71.55
PSPNet^48^	86.15	77.53	67.73	75.87
DANet^49^	89.16	81.14	72.19	80.65
DeepLab V3+^50^	89.08	**85.36**	73.12	80.57
A2-FPN^51^	89.12	82.61	73.59	80.72
MFCA-Net (ours)	**90.94**	84.77	**76.77**	**83.58**

**Figure 3:**
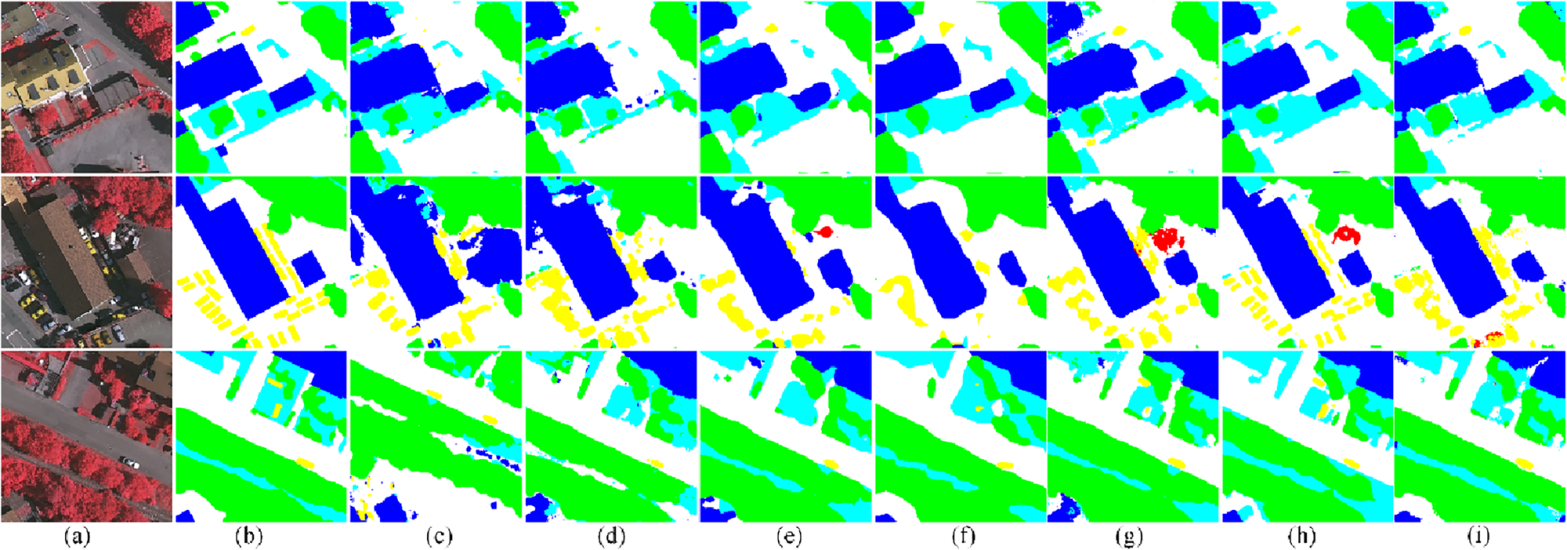
Visualization of the results of the Vaihingen testing set: (**a**) image (**b**) ground truth, (**c**) SegNet^47^, (**d**) U-Net^26^, (**e**) PSPNet^48^, (**f**) DANet^49^, (**g**) DeepLab V3+^50^, (**h**) A2-FPN^51^, and (i) Our proposed approach. (This figure was drawn by Visio 2021, which can be available at https://www.microsoftstore.com.cn/software/office/visio-standard-2021, The visualization was achieved in Visdom under the PyTorch framework. Vaihingen can be available at https://www.isprs.org/education/benchmarks/UrbanSemLab/2d-sem-label-vaihingen.aspx).

The visual inspection is presented in Fig. 3. We randomly select three samples and predict the pixel-wise label. Among all the methods compared, the MFCA-Net method has the greatest impact on vehicle recognition. For easily confused low vegetation and trees, the proposed MFCA-Net has a more accurate boundary delineation.

### Experiments on GID

Table 3 shows the experimental results of various methods in GID. The results show that the segmentation accuracy of SegNet and U-Net is relatively low; The segmentation accuracy of PSPNet and DANet is close. DeepLab V3+ has the highest accuracy among these six models, while A2-FPN segmentation accuracy is only higher than SegNet and U-Net. Analyzing the reasons, the variance between woodland and grassland classes is slight, and the proportion of woodland, grassland, and Buildings is also tiny, resulting in low segmentation accuracy for all three categories. After the proportion weighting calculation, the overall accuracy index was lowered. For datasets with slight inter-class variance, the segmentation accuracy of A2-FPN is low. The MFCA Net proposed in the paper outperforms the best DeepLab V3+ in all indicators. PA, MPA, MoU, and FWIoU indicators are 2.60%, 5.19%, 4.51%, and 3.86% higher than DeepLab V3+.

**Table 3:** Results on GID.

Method	PA	MPA	MIoU	FWIoU
SegNet^47^	65.40	66.44	48.53	48.56
U-Net^26^	67.11	62.71	49.25	50.37
PSPNet^48^	79.86	72.79	62.13	66.69
DANet^49^	79.67	79.54	64.62	66.48
DeepLab V3+^50^	82.77	80.50	69.43	70.82
A2-FPN^51^	63.62	74.47	53.52	59.36
MFCA-Net (ours)	85.37	85.69	73.94	74.68

**Figure 4:**
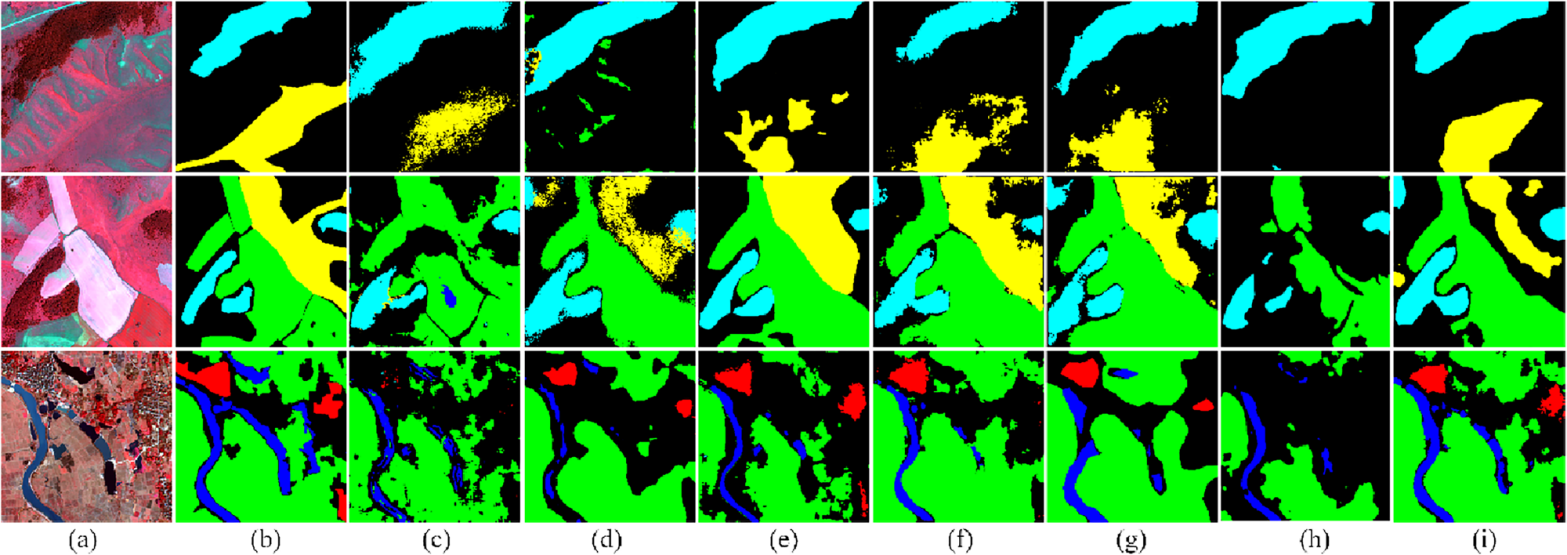
Visualization of the results of the GID testing set: (**a**) image, (**b**) ground truth, (**c**) SegNet^47^, (**d**) U-Net^26^, (**e**) PSPNet^48^, (**f**) DANet^49^, (**g**) DeepLab V3+^50^, (**h**) A2-FPN^51^, and (**i**) Our proposed approach. (This figure was drawn by Visio 2021, which can be available at https://www.microsoftstore.com.cn/software/office/visio-standard-2021, The visualization was achieved in Visdom under the PyTorch framework. GID can be available at https://www.cvmart.net/dataSets/detail/765?channel_id=op10&utm_source=cvmartmp&utm_campaign=datasets&utm_medium=article).

For qualitative evaluation, three samples of the GID testing set are predicted and illustrated in Fig. 4. In the dataset, the promotion of grassland is tiny; SegNet, U-Net, and the A2-FPN proposed in 2022 have poor recognition performance on grassland. A2-FPN did not perform as well as expected in identifying cultivated land and woodland. Compared with the other six models, the proposed MFCA-Net has better recognition performance for all classifications and smoother segmentation boundaries.

### Ablation study

The ablation study is implemented under the same hyperparameters and runtime environment. As presented in Table 4, the MPA and MIoU are collected to analyze the effects. We first list the segmentation accuracy on MobileNet V2 as the baseline network. Next, we investigated how IMV2 would influence the detection performance. It was observed that the MPA index improved by 4.08% and 5.39%, respectively, on the two datasets. On the MIoU index, the segmentation accuracy has been improved by 2.23% and 1.99%, respectively. Similarly, the performance of the MFDF module was verified. The MPA index increased by 2.51% and 3.96%, respectively; the MIoU index improved by 1.58% and 1.54%, respectively.

**Table 4:** Result of the ablation study.

Moudule	Vaihingen (MPA/MIoU)	GID (MPA/MIoU)
Baseline	78.18/72.96	76.34/70.4
IMV2	82.26/75.19	81.73/72.39
IMV2 + MFDF	84.77/76.77	85.69/73.93

**Figure 5:**
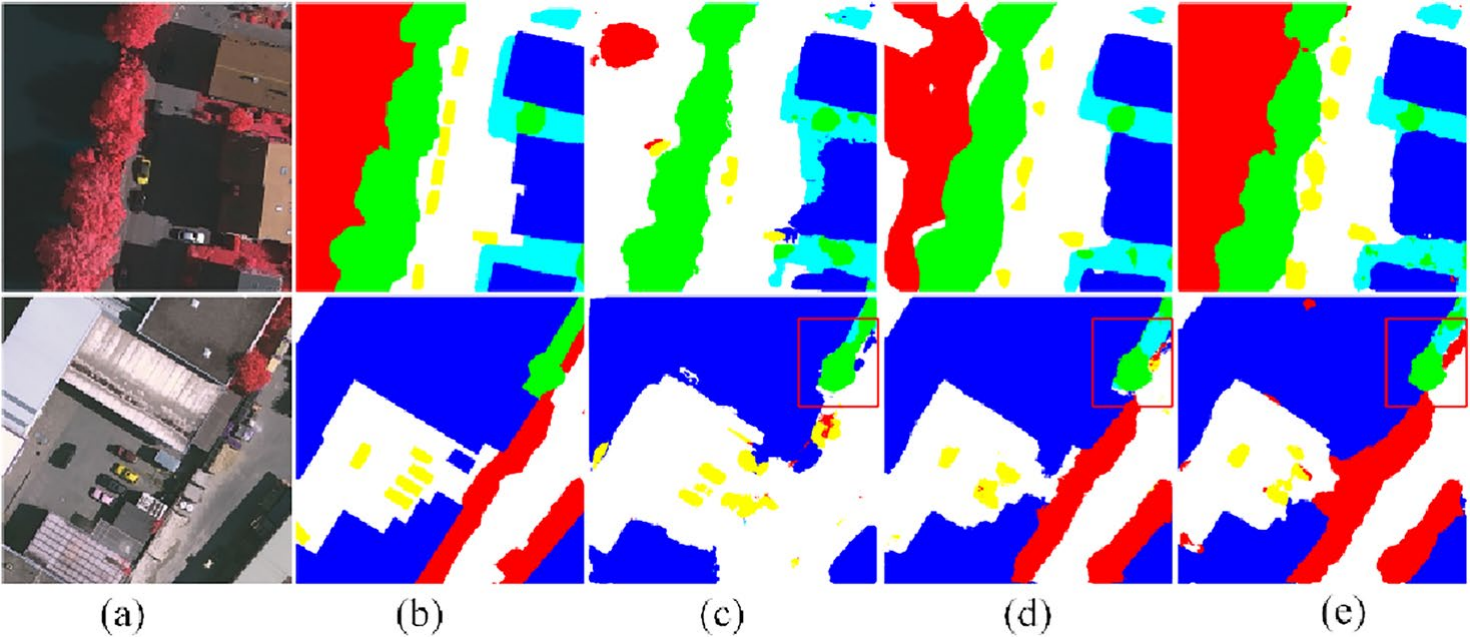
Visualization of the effect of IMV2 and MFDF on Vaihingen: (**a**) image (**b**) ground truth, (**c**) baseline, (**d**) IMV2, and (**e**) IMV2 + MFDF (this figure was drawn by Visio 2021, which can be available at https://www.microsoftstore.com.cn/software/office/visio-standard-2021, The visualization was achieved in Visdom under the PyTorch framework. Vaihingen can be available at https://www.isprs.org/education/benchmarks/UrbanSemLab/2d-sem-label-vaihingen.aspx).

Figure 5 shows the performance of IMV2 and MFDF by randomly selecting two images for visualization. The first two columns are input images and ground truth. The third column is the performance of the primary network, and the effect is not satisfied for the small proportion of clutter marked in red and cars marked in yellow. The fourth column shows the significant improvement after replacing MobileNet V2 with IMV2. In contrast, the last column shows the segmentation performance after continuing to add the MFDF module, which has better recognition performance for small samples and small target objects and is closer to the ground truth.

**Figure 6:**
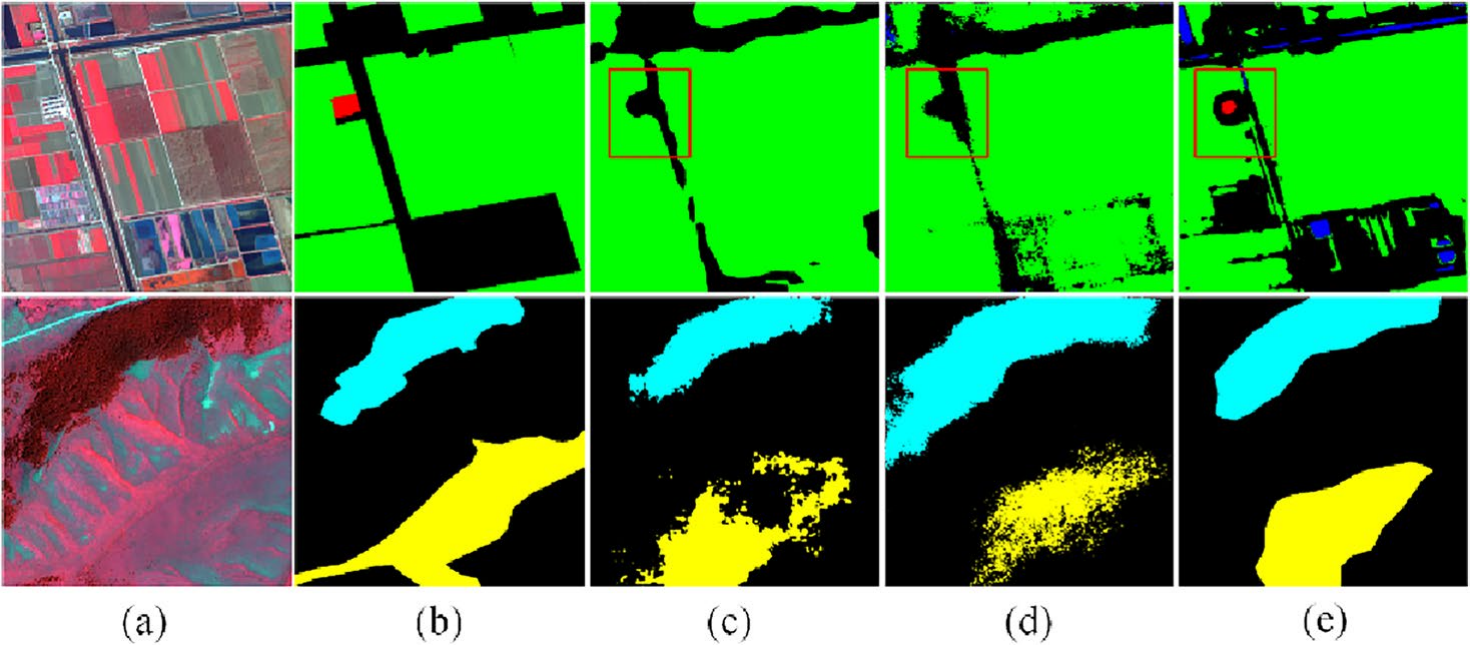
Visualization of the effect of IMV2 and MFDF on GID: (**a**) image, (**b**) ground truth, (**c**) baseline, (**d**) IMV2, and (**e**) IMV2 + MFDF (this figure was drawn by Visio 2021, which can be available at https://www.microsoftstore.com.cn/software/office/visio-standard-2021, The visualization was achieved in Visdom under the PyTorch framework. GID can be available at https://www.cvmart.net/dataSets/detail/765?channel_id=op10&utm_source=cvmartmp&utm_campaign=datasets&utm_medium=article).

Figure 6 shows the segmentation effect of IMV2 and MFDF on the GID dataset. The above figure did not identify the buildings marked in red based on the basic network and, after adding IMV2, ultimately identified the buildings after adding the MFDF module. It is difficult to distinguish between woodland in blue and grassland in yellow. The recognition effect is improving with the increase of IMV2 and MFDF modules.

## Conclusion

This paper proposes a novel MFCA-Net to improve semantic segmentation performance with RSI. The analysis introduced the channel attention module into the feature extraction network's shallow and deep feature maps, respectively. Moreover, a two-dimensional activation function FReLU that can obtain context information is adopted. After deep feature extraction, the MFDF module was designed. The upsampling process fused the three branches of the shallow feature map of the backbone network. The proposed MFCA-Net achieved better performance and higher detection accuracies than the state-of-the-art methods. The advantages of the proposed MFCA-Net can be briefly summarized as follows: (1) MFCA-Net obtained advanced semantic segmentation results. The experimental results indicate that MFCA-Net outperformed six widely used semantic segmentation methods in the visual observation and quantitative evaluation criteria. (2) The proposed MFCA-Net may achieve quick and effective learning performance and be quickly promoted in practical engineering applications. The findings on the relationship between the loss value and epoch indicate the temporary learning effect of MFCA-Net. These characteristics are acceptable and even preferred in practical applications. In our future studies, we plan to collect large-area datasets with other change detection methods and apply the proposed network to test its robustness and adaptability further.

## Data Availability

The datasets used or analyzed during the current study are available from the corresponding author on reasonable request.
